# Future Trend in Minimally Invasive Surgery: Single Port, Minilaparoscopy, and NOTES

**DOI:** 10.1155/2012/575301

**Published:** 2012-12-30

**Authors:** Luigi Boni, Paul G. Curcillo, Silvana Perretta

**Affiliations:** ^1^Minimally Invasive Surgery Center, University of Insubria, 20100 Varese, Italy; ^2^Minimally Invasive Surgical Initiatives and Development, Fox Chase Cancer Center, Philadelphia, PA 19111, USA; ^3^IRCAD, 67000 Strasbourg, France

Over the last few years, we assisted to an unstoppable quest for technical improvements in order to reduce even more the surgical trauma in the field of minimally invasive surgery. natural orifice transluminal endoscopic surgery (NOTES), single port surgery, and minilaparoscopy seem to answer the needs for reducing the trauma related to the “access” to the human body.

Among these techniques NOTES is, by no means, the most appealing and promising in terms of minimizing the access to the abdominal cavity; nevertheless, while single port and even more minilaparoscopy are currently applied worldwide in the routine clinical setting, for NOTES there are still several limitations, and it is often used only in clinical trials or experimental models, and, for most of the cases, it is performed under laparoscopic control.

On the other hand, there is no doubt that the current scientific literature is lacking robust data, and all these new technologies are still under evaluation to demonstrate that the reduction of surgical trauma in comparison to standard laparoscopy can justify the extra costs as well and the increase of operative difficulties.

In this special issue, ten original articles, reviews, and case reports have been selected because they described different experiences and modalities for what could be called “ultra” minimally invasive surgery.

As for NOTES not only a comprehensive review on current “state of the art” of NOTES in humans is published but also a paper describing results of a survey regarding patinets' perception on NOTES. As for specific clinical applications of NOTES three articles are focused on the approach to the spine and the role of transesophageal and transanal surgeries.

Readers will also find different papers regarding technique and results of single port surgery for colorectal, pancreatic, ileal resection as well as for cholecystectomy.

Finally, a well-documented technical note on applications of minilaparoscopy during colorectal resection is also presented.

*Luigi Boni* *Luigi Boni* *Paul G. Curcillo* *Paul G. Curcillo* *Silvana Perretta* *Silvana Perretta*

